# Serum neurofilament light chain in chronic inflammatory demyelinating polyneuropathy

**DOI:** 10.1002/brb3.2084

**Published:** 2021-02-22

**Authors:** Tomohiro Hayashi, Takamasa Nukui, Jin‐Lan Piao, Tomoyuki Sugimoto, Ryoko Anada, Noriyuki Matsuda, Mamoru Yamamoto, Hirofumi Konishi, Nobuhiro Dougu, Yuji Nakatsuji

**Affiliations:** ^1^ Department of Neurology Faculty of Medicine University of Toyama Toyama Japan; ^2^ Graduate School of Data Science Shiga University Shiga Japan

**Keywords:** biomarker, chronic inflammatory demyelinating polyneuropathy, neurofilament light chain

## Abstract

**Objectives:**

Neurofilament light chain (NfL) levels have been suggested as reflecting axonal damage in various inflammatory and neurodegenerative disorders, including acquired peripheral neuropathies. We aimed to investigate if serum NfL (sNfL) levels can be a biomarker of disease activity and treatment response in patients with chronic inflammatory demyelinating polyneuropathy (CIDP).

**Materials and methods:**

The sNfL levels of eleven newly diagnosed patients with CIDP were retrospectively assayed and compared with seven healthy volunteers. The levels were assayed before and after intravenous immunoglobulin treatment in patients with CIDP and were also assayed in the remission period.

**Results:**

Baseline sNfL levels in patients with CIDP before treatment were significantly higher than those in healthy controls. The levels significantly decreased overtime after one month of treatment and in remission period. There were significant negative correlations between the sNfL levels and the disease duration (the interval between the onset of the disease and the time of sampling), and weak correlations between the sNfL levels and overall neuropathy limitations scale.

**Conclusions:**

sNfL may be a potential biomarker reflecting the disease activity in patients with CIDP.

## Graphical abstract

Serum neurofilament light chain (sNfL) levels were assayed in patients with newly diagnosed chronic inflammatory demyelinating polyneuropathy (CIDP). We found that the levels in CIDP patients were significantly higher than those in healthy controls, and that the levels significantly decreased over time after treatment and in remission period. sNfL may be a potential biomarker reflecting the disease activity in patients with CIDP.

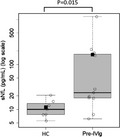

## 1 INTRODUCTION

Chronic inflammatory demyelinating polyneuropathy (CIDP) is the most common immune‐mediated neuropathy with a prevalence ranging from one to nine per 100,000 (Dalakas, 2011). CIDP typically presents with a progressive or relapsing course for more than eight weeks with both distal and proximal motor and sensory deficits. However, it is composed of clinically heterogeneous disorders with different phenotypic variants. According to the European Federation of Neurological Societies/Peripheral Nerve Society (EFNS/PNS) Guideline, several variants such as multifocal acquired demyelinating sensory and motor (MADSAM or Lewis‐Sumner syndrome), pure motor or sensory, and distal acquired demyelinating symmetric (DADS) polyneuropathy are included in CIDP (Van den Bergh et al., 2010). Randomized controlled trials have demonstrated that approximately two‐thirds of patients with CIDP improve with corticosteroids, immunoglobulins, and plasmapheresis. Maintenance therapy with intravenous immunoglobulin (IVIg) can induce sustained remission and prevent further axonal loss (Dalakas, 2011). Although a few biomarkers were reported to treatment response to IVIg such as the inhibitory FcγRIIb on B cells and transient axonal glycoprotein‐1 (TAG‐1), no established biomarkers indicative of disease activity or predictive of therapeutic responses for patients with CIDP have been reported (Iijima et al., 2009; Tackenberg et al., 2009).

Neurofilaments are the primary cytoskeletal proteins of neurons in both the central nervous system (CNS) and the peripheral nervous system (PNS), and form a lattice comprising neurofilament light (NfL), medium, and heavy chains (Perrot et al., 2008). As neuronal damage leads to the release of these proteins into the cerebrospinal fluid (CSF) and plasma, increased concentrations of NfL have been reported in neurological diseases such as amyotrophic lateral sclerosis (ALS), multiple sclerosis (MS), and Alzheimer disease (AD) (Disanto et al., 2017; Mattsson et al., 2017; Rosengren et al., 1996). In addition, NfL has recently been recognized as a diagnostic and prognostic biomarker of such neurological disorders (Bridel et al., 2019; Gaetani et al., 2019; Khalil et al., 2018; Zetterberg, 2016). An increase in the levels of sNfL has been reported recently with regard to peripheral neuropathy, and sNfL is suggested to be a novel biomarker of disease activity in patients with CIDP and Guillain–Barre syndrome (Altmann et al., 2020; Bischof et al., 2017; Gwen et al., 2019; Mariotto et al., 2018; Sandelius et al., 2018).

In this report, we assayed sNfL in patients with treatment‐naive CIDP before and after treatment with IVIg and also during remission. Correlations between sNfL levels and disease activity, treatment responses, and clinical features including laboratory data and neurophysiological findings were also investigated.

## 2 MATERIALS AND METHODS

### 2.1 Patients

Eleven patients who have been diagnosed with definite CIDP for the first time according to the EFNS/PNS 2010 criteria were studied retrospectively (Van den Bergh et al., 2010). MADSAM was defined as a typical mononeuropathy multiplex or asymmetry of symptoms, which was determined as differences in muscle strength by one or more Medical Research Council (MRC) scales in the homonymous muscles (Kuwabara et al., 2015). Demographic and clinical features, including disease duration and clinical severity assessed by the overall neuropathy limitations scale (ONLS), were collected (Graham & Hughes, 2006; Kuitwaard et al., 2015). Seven age‐matched healthy controls (HC) without a medical history of neurological diseases were included (Table 1). Patients were categorized into the following three groups: 1. Pretreatment group: newly diagnosed patients with CIDP starting treatment with IVIg for the first time (*n* = 11). 2. Post‐treatment group: patients with CIDP one month after the first treatment with IVIg (*n* = 7). 3. Remission group: patients in long‐term remission who had been off treatment or oral prednisolone or immunosuppressant for longer than six months (*n* = 9). This study was performed with the approval of the Ethics Committee of the University of Toyama (approval No. 29–32). Written informed consent was obtained from all patients.

**TABLE 1:** Clinical characteristics of patients with CIDP and HC

	CIDP patients			HC
	Pre‐IVIg	Post‐IVIg	Remission	
*n* (Male/Female)	11 (5/6)	7 (3/4)	9 (5/4)	7 (4/3)
Age, year; median (IQR)	59.6 (53.5–71.1)		61.8 (53.8–72.0)	58.0 (46.0–62.0)
Disease duration, week; median (IQR)	16.0 (8.0–28.0)		81.0 (72.9–153.0)	
CIDP subtype, *n*				
Typical:Atypical	7:4	4:3	6:3	
Combined treatment				
Prednisolone	*n* = 1 (2.5 mg)	*n* = 2 (2.5–10 mg)	*n* = 3 (5–10 mg)	
Serum NfL value, pg/ml; median (IQR), mean (±*SD*)	23.6 (18.0–169.1), 166.6 (337.8)	23.7 (17.3–170.5), 196.0 (345.8)	12.4 (10.2–27.9), 33.3 (42.1)	10.1 (8.2–13.6), 12.2 (6.3)
CSF NfL value, pg/ml; median (IQR)	826.5 (638.2–1329.9)			
ONLS; median (IQR)^a^	5.0 (3.0–6.0)	3.0 (1.5–5.5)	2.0 (1.0–3.0)	
grip strength, kilogram; mean (±*SD*)^a^	12.3 (8.3)	17.1 (10.2)	22.4 (8.3)	
MRC sum score; median (IQR)^a^	54.0 (52.5–56.5)	60.0 (54.5–60.0)	60.0 (60.0)	
Summated distal CMAP negative peak area, mVms; median (IQR)	30.3 (24.2–61.1)	32.7 (24.1–41.6)	56.7 (49.0–58.5)	

Abbreviations: CIDP, chronic inflammatory demyelinating polyneuropathy; CMAP, compound muscle action potential; CSF cerebrospinal fluid; EFNS/PNS, European Federation of Neurological Societies/Peripheral Nerve Society; HC, healthy control; IQR, interquartile range; IVIg, intravenous immunoglobulin; MADSAM, multifocal acquired demyelinating sensory and motor; MRC sum score, Medical Research Council sum score; NfL, neurofilament light chain; ONLS, Overall Neuropathy Limitations Scale.

^a^ The levels of ONSL (*p* =.003), grip strength (*p* =.0016), MRC sum score (*p* =.0010) significantly improved overtime (Mixed‐effect Regression model (repeated‐ANOVA trend test)).

### 2.2 Treatment

Eleven patients with CIDP received IVIg therapy. IVIg was administered using a conventional protocol (0.4 g/kg for 5 days). Treatment was considered effective when the symptoms improved by one or more on ONLS within four weeks after IVIg treatment.

### 2.3 Neurophysiological study

All patients underwent nerve conduction studies to confirm the diagnosis of CIDP. As an electrophysiological proxy marker of axonal damage, we used the lowest (left or right) summated distal compound muscle action potential (CMAP) negative peak area of the median, ulnar, and tibial nerves (Gwen et al., 2019).

### 2.4 NfL assay

Serum and CSF samples were centrifuged at room temperature, aliquoted in polypropylene tubes within 1 hr of collection, and then stored at −80°C until use. The concentration of NfL protein was determined in duplicates by investigators blinded to the clinical data using an HD‐1 immunoassay analyzer (Quanterix, Simoa, Lexington, MA, the United States of America), which runs ultra‐sensitive paramagnetic bead‐based enzyme‐linked immunosorbent assays.

### 2.5 Statistical analyses

A rank‐based nonparametric test (Mann–Whitney's U test) and Welch's *t* test were used for the patients with CIDP and controls, correlations were determined using nonparametric Spearman's correlation coefficient, and the corresponding plots were drawn on logarithmic scale. Longitudinal time‐effects were analyzed by trend test based on linear mixed regression model. Statistically significant differences were determined at a 5% level of probability. The statistical analysis was performed using JMP^®^14 (SAS Institute Inc.), or R version 4.0.2 (R Core Team, R Foundation for Statistical Computing).

## 3 RESULTS

**FIGURE 1:**
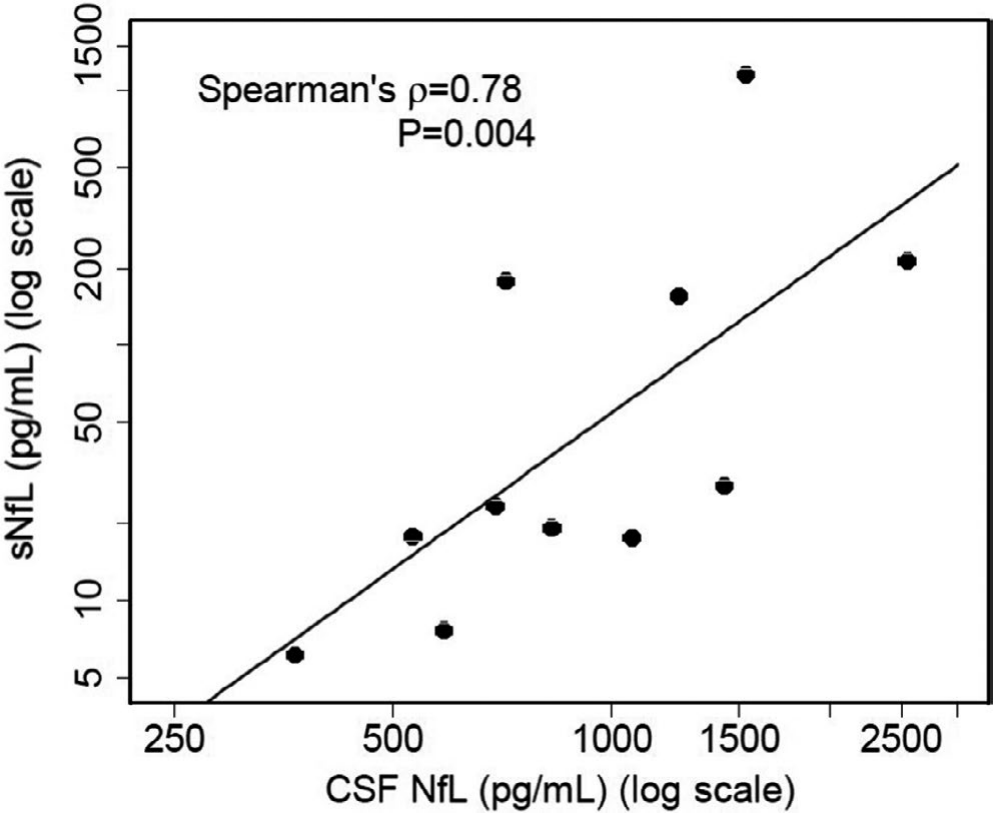
Correlation between the serum and the cerebrospinal fluid (CSF) neurofilament light chain (NfL) levels in patients with treatment‐naïve CIDP. The sNfL levels significantly correlated with CSF NfL. Correlation is presented with Spearman's ρ and *p*‐value

**FIGURE 2:**
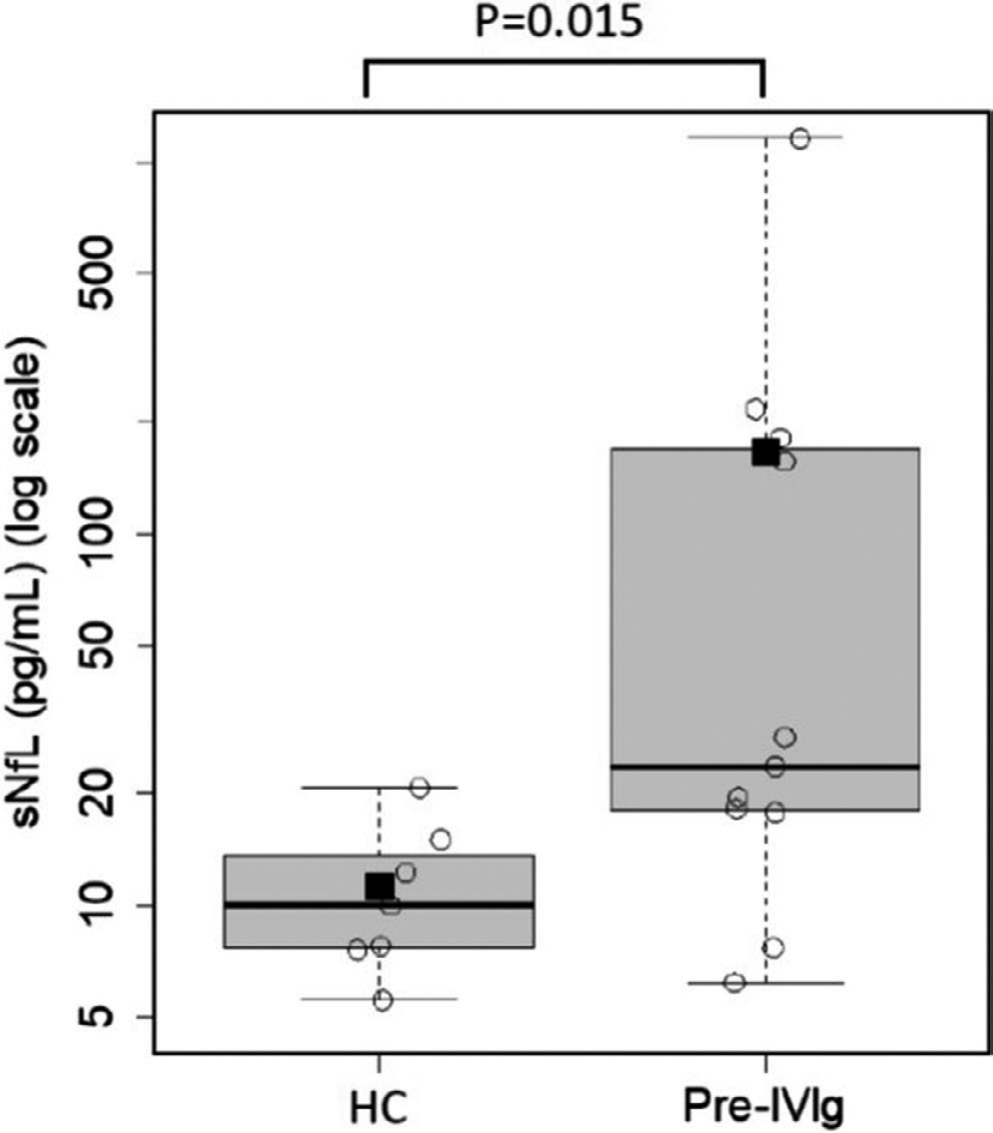
sNfL levels in patients with treatment‐naïve CIDP (Pre‐IVIg) and in healthy controls (HC). Closed squares and horizontal bars represent group means and group medians, respectively. The sNfL levels were significantly higher in the CIDP pretreatment group than in HC. Correlation is presented with Welch's *t* test and *p*‐value

**FIGURE 3:**
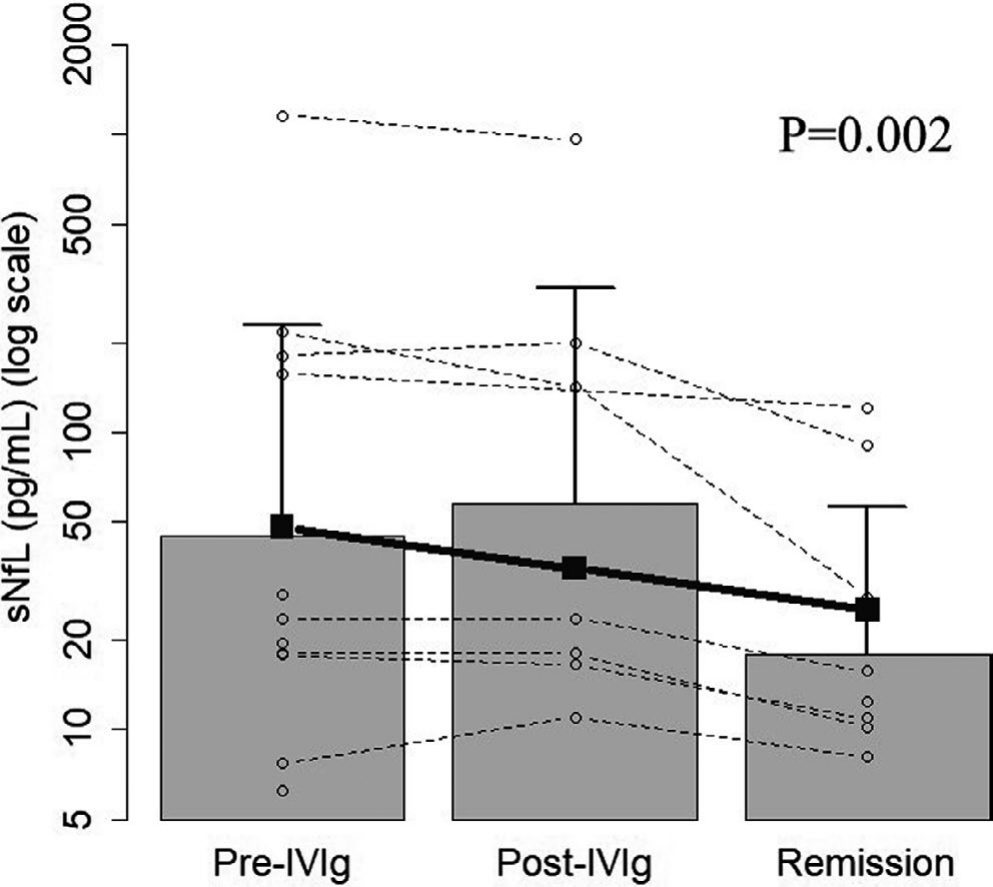
Changes in sNfL levels in each individual patient with CIDP at different disease phases of Pre‐IVIg, Post‐IVIg, and remission. The sNfL levels significantly decreased overtime after one month of treatment and in remission period. Closed squares represent means. Longitudinal time‐effects are presented with trend test based on linear mixed regression model and *p*‐value

Eleven patients with CIDP and seven healthy controls were enrolled in this study. Patient demographics are shown in Table 1. The cohort consisted of patients with typical CIDP (*n* = 7), MADSAM (*n* = 3), and pure sensory (*n* = 1). The median disease duration before blood sampling was 16.0 weeks (interquartile range [IQR]:8.0–28.0). No patients received prior therapy for CIDP, though one patient had been received 2.5 mg of prednisolone for the treatment of idiopathic thrombocytopenic purpura at pre–IVIg sampling. The median ONLS of the pretreatment, post‐IVIg, and remission groups were 5.0 (IQR: 3.0–6.0), 3.0 (IQR: 1.5–5.5), and 2.0 (IQR: 1.0–3.0), respectively (Table 1). The sNfL levels were significantly correlated with cerebrospinal fluid NfL (CSF NfL) at pre‐IVIg sampling (*n* = 11, Spearman's rho = 0.78, *p* =.004) (Figure 1). The sNfL levels were significantly higher in the CIDP pretreatment group (mean 166.6 ± 337.8 (*SD*) pg/mL than in the healthy controls (mean 12.2 ± 6.3 pg/ml) (Welch's *t* test, *p* =.015) (Figure 2). The sNfL levels significantly decreased overtime after one month of treatment and in remission period (Mixed‐effect Regression model (repeated‐ANOVA trend test, *p* = .002)) (Figure 3). The levels of ONLS, grip strength, and MRC sum score also significantly improved overtime after one month of treatment and in remission period (trend test based on linear mixed regression model ), ONLS: *p* = .0003, grip power: *p* = .0016, MRC sum score: *p* = .0010) (Table 1).

**FIGURE 4:**
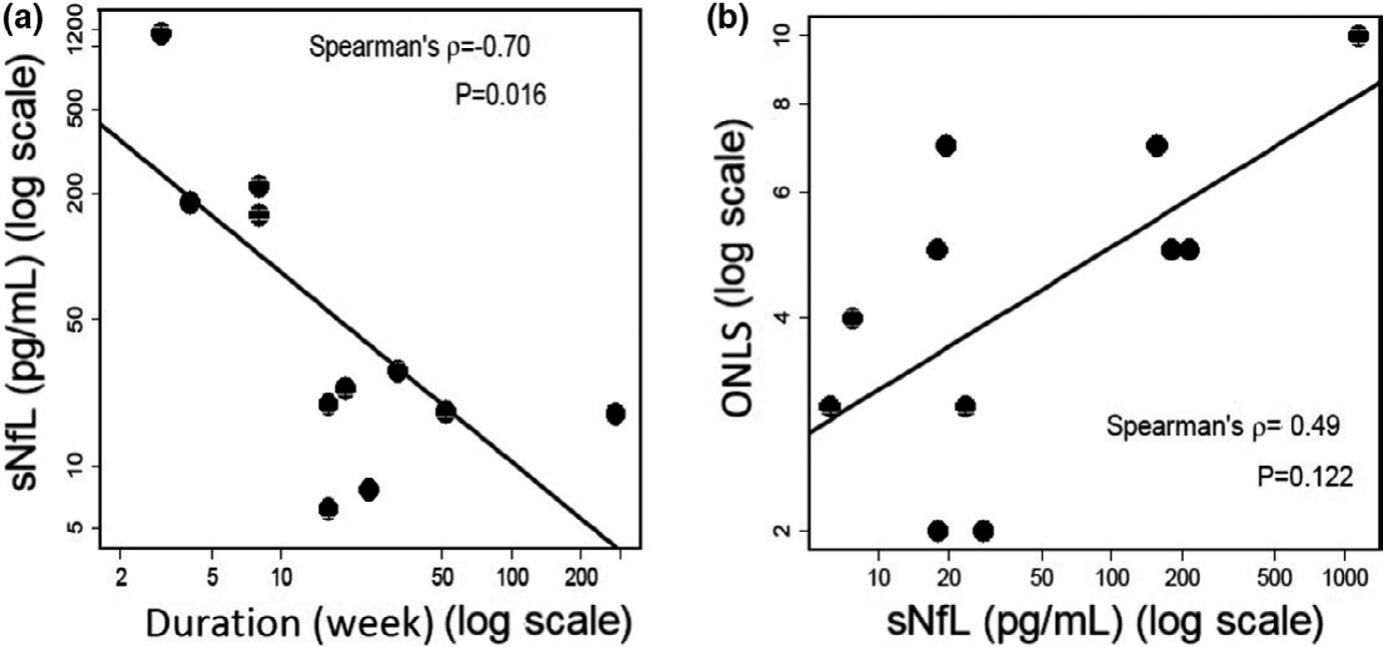
Correlation between the sNfL levels in patients with treatment‐naïve CIDP (Pre‐IVIg) and disease duration (the interval between new‐onset episode and serum collection; week) (a) and Overall Neuropathy Limitations Scale (ONLS) (b). A significant negative correlation was observed between sNfL and disease duration, and there was a correlation between sNfL and ONLS, though it was not significant. Correlation is presented with Spearman's ρ and *p*‐value

Next, we examined whether there were correlations between sNfL levels and disease activity, treatment responses, and clinical features, including laboratory data and neurophysiological findings. A significant negative correlation was observed between sNfL and disease duration (*n* = 11, Spearman's rho = −0.70, *p* = .016) (Figure 4a). There was a correlation between sNfL and ONLS, though it was not significant (*n* = 11, Spearman's rho = 0.49, *p* = .122) (Figure 4b). There were no correlations between the sNfL levels and CIDP subtype (typical/atypical), age, grip strength, MRC sum score, and summated distal CMAP negative peak area (Table 2).

**TABLE 2:** Correlation analyses between sNfL levels in treatment‐naïve patients with CIDP at pre‐IVIg and disease activity, treatment responses, and clinical features

		sNfL (Pre‐IVIg)
CIDP subtype, *n* typical : atypical	7:4	*p* = .257
Age, year^a^	59.6 (53.5–71.1)	*r* = 0.30 *p* =.370
Disease duration, week^a^	16.0 (8.0–28.0)	*r* = −0.70 *p* = .016
ONLS^a^	5.0 (3.0–6.0)	*r* = 0.49 *p* =.122
Grip strength; kg, mean (*SD*)	12.3 (8.3)	*r* = −0.31 *p* = .347
MRC sum score^a^	54.0 (52.5–56.5)	*r* = −0.38 *p* = .250
Summated distal CMAP negative peak area, mVms^a^	30.3 (24.2–61.1)	*r* = −0.28 *p* = .401

Abbreviations: CIDP, chronic inflammatory demyelinating polyneuropathy; CMAP, compound muscle action potential; IQR, interquartile range; IVIg, intravenous immunoglobulin; MRC sum score, Medical Research Council sum score; ONLS, Overall Neuropathy Limitations Scale; *SD*, standard deviation; sNfL, serum neurofilament light chain.

^a^ Median (IQR).

## 4 DISCUSSION

In the present study of patients with CIDP in three different phases, significant increase in the sNfL levels was observed in patients with treatment‐naïve CIDP who were supposed to have high disease activity. This result is in line with a previous report (Mariotto et al., 2018). The sNfL levels tended to decrease in the post‐IVIg and remission groups significantly compared to those in the pre‐IVIg group, which seems at least in part to reflect the treatment response. On the other hand, the timing of NfL measurement could influence its concentration, especially in relation to the latest exacerbation and treatment (Faravelli et al., 2020; Gaetani et al., 2019; Wurster et al., 2019). CSF NfL levels in patients with multiple sclerosis, for example, is thought to remain high for two to three months after relapse before dropping to lower levels after treatment, and this seems to apply to sNfL in CIDP as well (Gaetani et al., 2019). The levels of sNfL at post‐IVIg period might have been more significantly decreased if we took blood sample 2 to 3 months after treatments with IVIg. The significant increase in the levels of sNfL seems to imply an underlying severe neuroaxonal damage during the acute phase of patients with treatment‐naïve CIDP. It would be better to assay sNfL at more specific time points chronologically to reveal the dynamics of axonal damage.

The levels of sNfL reflect neuroaxonal damage, and treatment with IVIg could repair axonal damage (Khalil et al., 2018). However, no correlation between the levels of sNfL and summated distal CMAP negative peak area was detected. As the sNfL levels of patients in remission tended to be lower than those in the post‐IVIg group, a more extended observation period might be necessary to repair neuroaxonal damage and detect correlations between the sNfL levels and electrophysiological examination.

Recent studies have clarified that the mechanisms of neuropathy in patients with IgG4 autoantibodies against paranodal junction components such as neurofascin 155 (NF155) are different from those in conventional CIDP patients with macrophage‐induced demyelination (Koike et al., 2017; Koike & Katsuno, 2020). Although we did not examine anti‐NF155 antibody in our study, the levels of NfL may be higher in patients with this antibody than in those with conventional CIDP because severer axonal degeneration is suggested to occur in the former. The analysis based on the positivity of anti‐NF155 antibody is of interest.

This study had several limitations in that it was a hospital‐based cross‐sectional study, and the sample size was small.

In summary, our results suggest that sNfL may be a potential biomarker for assisting the diagnosis and evaluating the activity of CIDP. Further studies are needed to clarify the significance of sNfL as a biomarker for CIDP.

## CONFLICT OF INTEREST

The authors declare no conflicts of interest associated with this manuscript.

## AUTHOR CONTRIBUTION

T.H, T.N., J‐L.P, and Y.N. took part in the study design. R.A., N.M., M.Y., H.K., and N.D. performed the clinical evaluation. T.H. and T.N. analyzed the data. T.S. performed the statistical evaluation. All authors contributed to the interpretation of data. T.H. wrote the manuscript draft, which was critically revised and finally approved by all authors. Y.N. supervised the study.

### Peer Review

The peer review history for this article is available at https://publons.com/publon/10.1002/brb3.2084.

## Data Availability

The data that support the findings of this study are available from the corresponding author upon reasonable request.
